# Almond Tree Adaptation to Water Stress: Differences in Physiological Performance and Yield Responses among Four Cultivar Grown in Mediterranean Environment

**DOI:** 10.3390/plants12051131

**Published:** 2023-03-02

**Authors:** Ana Fernandes de Oliveira, Massimiliano Giuseppe Mameli, Luciano De Pau, Daniela Satta

**Affiliations:** Agris Sardegna, Agricultural Research Agency of Sardinia, Loc. Bonassai S.S. 291 Sassari-Fertilia—Km. 18600, 07100 Sassari, Italy

**Keywords:** *Prunus amygdalus Batsch cultivar*, ‘Arrubia’, ‘Cossu’, ‘Texas’, ‘Tuono’, water deficit acclimation, physiological behavior, crop performance, Mediterranean areas

## Abstract

Maximizing water use efficiency, yield, and plant survival under drought is a relevant research issue for almond-tree-growing areas worldwide. The intraspecific diversity of this species may constitute a valuable resource to address the resilience and productivity challenges that climate change poses to crop sustainability. A comparative evaluation of physiological and productive performance of four almond varieties: ‘Arrubia’, ‘Cossu’, ‘Texas’, and ‘Tuono’, field-grown in Sardinia, Italy, was performed. A great variability in the plasticity to cope with soil water scarcity and a diverse capacity to adapt to drought and heat stresses during fruit development were highlighted. The two Sardinian varieties, Arrubia and Cossu, showed differences in water stress tolerance, photosynthetic and photochemical activity, and crop yield. ‘Arrubia’ and ‘Texas’ showed greater physiological acclimation to water stress while maintaining higher yields, as compared to the self-fertile ‘Tuono’. The important role of crop load and specific anatomical traits affecting leaf hydraulic conductance and leaf gas exchanges efficiency (i.e., dominant shoot type, leaf size and roughness) was evidenced. The study highlights the importance of characterizing the relationships among almond cultivar traits that affect plant performance under drought in order to better assist planting choices and orchard irrigation management for given environmental contexts.

## 1. Introduction

The sweet almond tree (*Prunus amygdalus* Batsch, var. *dulcis*) is the result of domestication and cultivation of the wild plant, generally characterized by bitter seeds (*Prunus amygdalus* Batsch var. *amara*), from the Middle East [1,2,3]. It was introduced into Europe between the 6th and 5th centuries B.C. by the Greeks and Phoenicians. In Europe, the crop is distributed between 36° and 45° parallels, but it can be found even at higher north latitudes and is particularly widespread in Spain, Italy, Portugal, France, and Greece. Among the producing countries, the United States remains unchallenged at the top of the ranking, followed at distance by China, Turkey, Iran, India, and Northern Africa countries [4]. California almond cultivation remains steady as the world leader in the sector, due to the crop innovation that took place during recent decades, comprising the development of new soft-shelled varieties and the intensification of cultivation (e.g., planting density, irrigation, and mechanized harvesting) [5]. The strong expansion of almond crop growing areas over the last decade is also due to the availability of new rootstocks that allow cultivation in soils previously considered unsuitable [6]. Until the 1970s, Italy was the world leader in almond production, strongly stimulated by domestic demand and local confectionery tradition. However, the strong competitiveness of Californian and Australian productions in the following decades led to the abandonment of cultivation in many traditional almond-growing areas of Italy, characterized by old and poorly productive almond orchards [7]. Though local varieties still persist in much of today’s cultivation [8], partly because of their specific compositional attributes [9], the plantation of new intensive and super-high-density [5], irrigated orchards with a prevalence of self-fertile varieties are currently expanding. The growing global market demand has much contributed to this cultural intensification, prompted by the affirmation of the web market and by consumer recognition of the high nutritional and nutraceutical value of almonds [10]. Despite the biodiversity of almond varieties in the Mediterranean basin [2,3,11], today, almond production is dominated by a few commercial varieties [12]. In Italy, only a few local varieties are still cultivated, while 329 cultivar are referred and characterized (about 140 varieties in Apulia, 100 in Sicily, 40 in Sardinia, and 13 in Abruzzo). In addition, most Italian almond orchards are planted with self-incompatible and early-blooming varieties, exploiting a variety of gametophytic compatibility for cross-pollination [6,13]. Usually, these orchards are grown using the traditional systems, grafted onto rootstocks of medium to high vigor, with low to medium planting densities [14], unirrigated, or applying emergency and low re-watering supplies with drip irrigation [7,15].

The key to success in the new intensive almond orchards was strongly supported by the selection of blooming and self-compatible cultivar, to guarantee effective pollination, with low susceptibility to chlorosis and higher resistance to pests and diseases. In addition, the selection of new dwarfing rootstocks, with greater tolerance to asphyxia and chlorosis, enabled higher planting densities [16]. However, to ensure high yields and economic sustainability of such plants, the use of low-deficit irrigation strategies during periods of greatest water needs and scarcity becomes essential [17,18,19]. Furthermore, the impact of ongoing global warming challenges the sustainability and survival of new intensive cropping systems [17,20]. Despite almond’s drought resistance, it has poor salt stress tolerance [21,22], and prolonged water scarcity during fruit developmental stages, concomitant with high atmospheric evaporative demand, severely compromises annual yields [23,24]. In Mediterranean arid or semi-arid climates, seasonal drought combined with heat waves and limited irrigation supplies result in a rapid decline in plant water status. Consequently, intense defoliation and tissue dehydration occur, which can trigger plant mortality [17,25]. In fact, almonds acclimate better to gradual water stress increases [26,27].

For these reasons, it is important to understand the complex set of mechanisms triggered by plants in response to water stress and to investigate varietal physiological and morphological differences that may affect orchard yield, water use efficiency, and translocation from source to sink organs [28,29]. Such studies can help evaluate crop adaptation to arid and semi-arid environments. In this study, we carried out a comparative evaluation of plant physiological and productive behavior and of the acclimation to water stress conditions of four almond tree varieties field-grown in Sardinia: two among the main varieties of national and/or international agroeconomic interest, Tuono and Texas; two local varieties cultivated for their agronomic and industrial characteristics, Arrubia and Cossu [9,12]. The genetic and phenotypic richness of this species [1,3,8,9,12,13,30,31] constitutes a valuable resource for genetic breeding and varietal selection, to enable the combination of varietal traits that result in more effective stress-response strategies with the ability to maintain high yield and quality standards. Understanding the determinants of higher or lower stress tolerance of a given cultivar will, thus, increase resilience in order to address the challenges that climate change poses to the environmental and economic sustainability of almond production [32,33,34,35]. In this study, we comparative analyzed plant water status, leaf gas exchange, and photochemical performance from the fruit development stage to harvest, together with crop yield compounds and fruit and leaf morphological traits during two consecutive growing seasons, 2020 and 2021. This study allowed for characterizing varietal acclimation responses to the environmental context and to identify major variety-related factors that lead to different yield performance, stress tolerance, and water use efficiency under a deficit irrigation.

## 2. Materials and Methods

### 2.1. Orchard and Experimental Site

The trial was conducted during the seasons 2020 and 2021 at the experimental field-grown almond cultivar collection of the Agris Sardegna agency, in Sassari, Italy (40°47′50.12″ N; 8°28′32.22″ E, about 53 m a.s.l.). This agency conserves 40 autochthonous almond varieties and 29 national and international varieties, comprising Sardinian native, Apulian, Sicilian, and also Californian varieties, all grafted onto GF-677 rootstock. 

Following a randomized block experimental design, the orchard was planted in 2009, with trees distributed in 23 horizontal rows and a planting distance of 5 m × 5 m (400 plants ha^−1^). Each row contains 5 plants of three different varieties, distributed along 3 consecutive blocks. Plants were pruned to a classic free-vase with 3–4 branches at 80 cm from the soil (Figure S1). Pruning was performed yearly to retain the vase shape and to reduce excessive vegetation density in the inner and upper parts of the canopy, therefore allowing for efficient mechanical harvesting. The soil is calcareous clayey-loam with a 1.5–2 m depth, approximately. Soil texture, organic matter, pH, and hydraulic properties were determined using the gravimetric method. The soil samples were collected at 0–20 m and 20–50 cm depth, in 3 different locations along each block (Table A1). Drip irrigation was supplied by a subsurface double dripline, located 40 cm aside from the plant row and buried at 20 cm depth, in both sides of the plant row. Natural cover crop was managed with spring mowing in the inter-rows and chemical weed control under the tree canopy. In alternate years, the topsoil was tilled with subsurface harrowing. The physiological and yield performance under water stress of the two main local varieties, Arrubia and Cossu, were compared to those of two of the most common Italian and Californian cultivated varieties, Tuono and Texas, respectively. 

### 2.2. Cultivar Description

‘Arrubia’ is one of the most widely grown almond varieties in Sardinia [36]. The plant shows high vigor, moderately spreading growth habit, and high yield, blooming normally by mid-February mainly on one-year-old shoots. The hull is suffused red, the fruit shell is hard and porous, and the almond is oblong with medium to high size and weight, with a reduced percentage of double kernels or empty fruits (about 1%) and a shelled yield of nearly 30%. The plant shows fruiting consistency along the years, together with the higher yields and fruit quality compared to other local varieties, and also good industrial acceptance, especially for confectionery [1]. ‘Cossu’ is another cultivar widely grown in Sardinia [37,38]. The tree has a moderately spreading growth habit and low vigor, with late bloom (from the end of February to the beginning of March), both on spurs and one-year-old wood. The kernel, of medium size, has ellipsoidal form and the shell is hard and ovoid with medium porosity. The shelled yield is about 25%, with a low percentage of double kernel and empty shells, and ripening is moderately early. ‘Tuono’ is a variety native from Apulia and currently cultivated in the main Italian almond cropping areas and other European regions, due to its self-fertility and the good fruit characteristics [39]. The tree presents low vigor and expanded growth habit, with medium to late bloom, fructifying on both mixed shoots and spurs, and retaining medium yield level. The fruit is typically amygdaloidal with medium size, with a long elliptical kernel and early ripening, but with a high percentage of double kernels (about 15–20%) and alternating yield [40]. ‘Texas’ (syn. ‘Texas Mission’ syn. ‘Texas Prolific’ [41]) is a Californian variety quite well adapted to Mediterranean environments, with vigor and erect behavior and an open spreading canopy [42]. It is self-incompatible and it blooms late and quite gradually in the tree [41,42]. Thus, it can yield high production, with relatively late ripening. The fruit is medium sized with amygdaloidal form. The shelled yield is high and the kernel is medium sized and semi-hard with a relatively high percentage of double kernels (ca., 15–20%), therefore being mainly used for the processing ground almond industry.

Leaf length and width were measured on 15 fully expanded leaf samples collected from 3 plants of each variety [43]. A leaf area meter (LI-3100C, LI-COR Biosciences, Lincoln, NE, USA) was used to measure the area of each leaf replicate. The results are shown in Appendix A (Table A2).

### 2.3. Weather, Water Balance, and Irrigation Management

Meteorological data (air temperature, relative humidity, wind speed, solar radiation, and precipitation) were gathered hourly, during the 2 years of study, by a weather station (Pessl instruments: Werksweg 107, 8160 Weiz, Austria) located close to the experimental site (40°47′45.9132″ N; 8°28′33.8778″ E, 60 m a.s.l.). Potential evapotranspiration (ET0) was determined based on the Penman–Monteith method [44]. Then, cultural evapotranspiration (ETc) was estimated by applying seasonal crop coefficients (Kc) according to the growth stage: 0.75 from fruit set to pit hardening; 0.95 from nut growth until ripening; 0.85 from harvest to bud differentiation [45]. The variation in precipitation deficit (PD) was estimated from January until September, based on soil water content, field capacity, and the daily accumulated difference between precipitation and potential evapotranspiration. Irrigation was scheduled biweekly from May to September and, in 2021, the returned ETc was calculated based on the irrigation and precipitation amounts with respect to crop evapotranspiration during each phenological stage. The analysis of seasonal trends took into consideration also the hydro-climatological historical data and the agrometeorological reports of the regional meteoclimatic department [46,47].

### 2.4. Plant Water Status and Leaf Physiological Performance

Stem water potential (Ψ_stem_) was measured every 2 weeks on two exposed shoots, with adult leaves (10 replicates), facing the east and west sides and located at the middle height of the canopy, using a pressure chamber (Model 1000, PMS Instrument Company, Albany, NY, USA) [48]. About one hour prior to the measurements, the shoots were enclosed into aluminum-foil-coated plastic bags in order equilibrate the water status of the leaves with that of the shoot, thus allowing for bringing it closer to the actual water status of the photosynthetically active canopy. The Ψ_stem_ gives a good indication of both plant water uptake and transpiration status. In addition, it represents plant hydraulic conductivity and it is less affected by temporary variations in the atmospheric conditions, which would affect data if measured directly on the uncovered shoots [49]. In order to detect effective water deficit and irrigation needs, Ψ_stem_ was measured at midday, therefore representing the daily maximum atmospheric demand and water stress conditions. Based on the observation of stem water potential, the scheduled irrigation events were adjusted to maintain mild to moderate plant water status. At the same time, leaf gas exchange was monitored on two fully expanded leaves of each plant, from similar shoots of those chosen for Ψ_stem_, using a portable infrared gas analyzer (CIRAS-3, PP systems, Amesbury, MA, USA) assembled to a Parkinson leaf chamber. Net assimilation rate (Pn, μmol CO_2_ m^−2^ s^−1^), stomatal conductance (gs, mmol H_2_O m^−2^ s^−1^), transpiration rate (E, mmol H_2_O m^−2^ s^−1^), intrinsic water use efficiency (WUEi, μmol/mmol), instantaneous water use efficiency (WUE, μmol/mmol), and leaf temperature were measured on fully expanded leaves. Reference air was set with a CO_2_ concentration of 400 μmol mol^−1^ and a 55% relative humidity, and the photosynthetic photon flux density was standardized at 1000 μmol m^−2^ s^−1^ by coupling a LED light unit to the leaf chamber. Besides midday (13.00–14.00 h) Ψ_stem_ and photosynthetic activity, mid-morning (9.00–10.00 h) measurements of those physiological variables were also monitored on 4 days with clear sky conditions, during nut growth and ripening stages of each growing season of study. Simultaneously, the direct chlorophyll fluorescence of PSII was monitored in vivo on fully expanded leaves (10 replicates). After a dark adaptation period of 30 min, fluorescence transients (OJIP curve) were recorded using a Handy PEA fluorimeter (Hansatech Instruments Ltd., King’s Lynn, UK) after the application of actinic saturating red light (650 nm wavelength) of 3000 µmol m^−2^ s^−1^ single flashes, with the signal gain at 1.0 and a 30 s duration of each replicate. Rapid fluorescence kinetics from the minimum (Fo) to the maximum (Fm) fluorescence of dark-adapted leaves was collected. The fluorescence signals were recorded at a 10 µs time step, allowing for main and derived fluorescence variables calculation (Table 1) and, thus, for evaluation of the PSII performance during photochemical and thermal phases of the fluorescence transient [50,51]. 

### 2.5. Phenological Succession, Morphological Traits, and Yield Components

Phenological succession recorded on a weekly basis and fruit morphological traits and yield were monitored in the three replicate plants of each cultivar, following the CIAT (Alliance of Bioversity International and the International Center for Tropical Agriculture) almond descriptors [53]. Yield components, including hulled fruit, nut and kernel weight (g), kernel percentage, percentage of nuts without kernel, and morphological fruit traits, such as nut length (cm) and width (cm), kernel length (cm), width (cm), and double kernel percentage, were measured on a thirty-fruit sample of each replicate.

### 2.6. Statistical Analysis

Heat maps were applied on mean midday Ψ_stem_ values of each cultivar along the different phenological stages, using Excel conditional formatting, to show the magnitude of the variation in water status of each variety over the two years of study. All data were subjected to one-way analysis of variance (ANOVA) and the least significant difference (LSD) test, using the software SPSS 25.0 (SPSS Inc., Chicago, IL, USA), in order to compare means and detect significant differences among cultivar at the 95% confidence level. Two-way ANOVA and the LSD test were also carried out for Ψ_stem_, leaf gas exchange, and photochemical variables measured at mid-morning and midday, in order to compared means and to evaluate main significant influences of the cultivar and time of measuring, at a *p*-value of 0.05, as well as to detect interaction effects among these two main factors. Similarly, main and interaction effects of the cultivar and between-year differences were investigated in mean values of yield components. Furthermore, box-whisker plots were created using XLSTAT-Pro 2014 (Addinsoft Inc., New York, NY, USA), where horizontal lines represent the median, squares represent the mean, boxes represent the interquartile range (IQR), whiskers represent 1.5 × IQR, and crosses outside boxes represent the outliers. Additionally, after scaling and normalizing the data, principal component analysis (PCA) was performed on the cultivar main physiological variable measured at midday for which significant differences were detected (including Ψ_stem_, gs, WUEi, and WUE), during the two seasons of study. Pearson’s correlation was used to assess the similarity between variables and factors. Finally, yield and the physiological variables most directly related to plant water use efficiency were compared by plotting the cultivar and variables in a single PCA biplot. The resulting loading and score plots highlighted common variations and made it possible to summarize the strength of the correlations between each variable and cultivar and, thus, to highlight the main sources of physiological differences affecting yield, plant water status, and leaf gas exchange, in response to water stress of the four cultivar under the environmental conditions of the study.

## 3. Results

### 3.1. Orchard Phenology, Season Water Balance, and Plant Water Status

**Figure 1:**
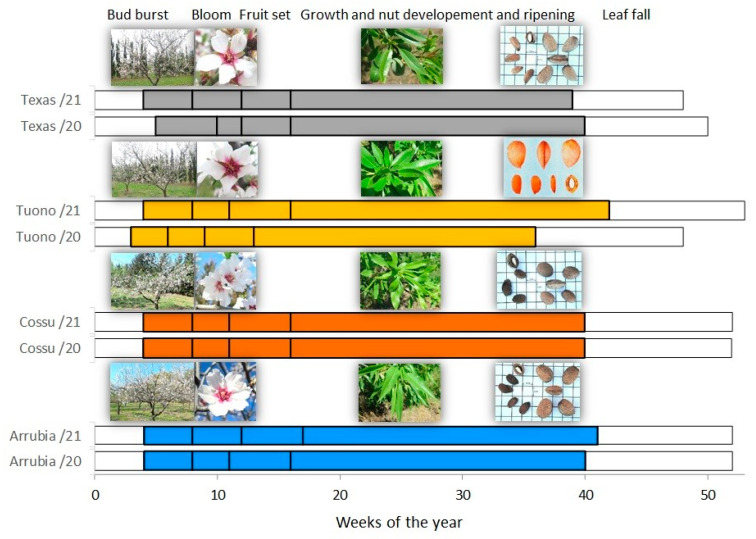
Diagram of the phenological courses of ‘Arrubia’, ‘Cossu’, ‘Tuono,’ and ‘Texas’ during the growing seasons 2020 and 2021 in the studied field collection orchard.

Figure 1 shows the phenological course of the four varieties along the two study seasons. From bud swelling until leaf fall, cultivar Arrubia and Cossu showed consistency in the overall cycle and in the single-stage duration between seasons: about 48 weeks in both seasons, with 3 to 4 weeks from bud burst to bloom, 5 weeks to fruit set, 24 weeks for fruit growth and ripening, and 11 to 12 weeks for leaf fall. The cycle duration was similar in ‘Texas’, with 48 weeks in 2020 and 47 weeks and 2021, but compared to the second season, bud burst occurred 2 weeks later in 2020 and took about 5 weeks (1 week longer), while the duration of bloom was half of that of 2021. In this variety, fruit set took place in 3 weeks in both seasons, and fruit growth and ripening were within 24 and 23 weeks, respectively. In ‘Tuono’, the cycle was anticipated in 2020 compared to 2021 and with the other varieties. Despite the similar overall duration (48 weeks), bud burst started earlier (in week 3), bloom and fruit set were completed within only 7 weeks instead of 8, and fruit growth and ripening took place within 23 instead of the 26 weeks of 2021. As a result, ripening was complete 4 weeks earlier in 2020.

**Figure 2:**
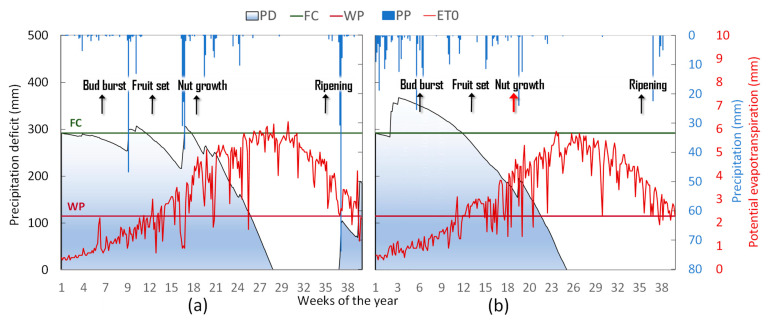
Patterns of precipitation deficit (PD), field capacity (FC), wilting point (WP), precipitation (PP), and potential evapotranspiration (ETo) during the growing seasons 2020 (**a**) and 2021 (**b**), from January to September.

The weather season 2020 was close to the climatic average in terms of the number of rainy days and accumulated precipitation [45], but the January–March quarter was very dry (Table 2 and Figure 2). Annual minimum temperatures were slightly above the climatic average while maximum temperatures were higher than the average and peaks above 40 °C were exceeded in July and August. The hydroclimatic balance (expressed by the difference between rainfall inputs and potential evapotranspiration) was characterized by prolonged deficit, particularly intense during the spring and summer months reversed only by September, thanks to abundant rainfall. Accumulated precipitation was much lower in 2021 (Figure 2), 482 mm against the 800 mm in 2020, and the average rainfall was below the climatological mean [46]. Scarce rain events from April to September resulted in a very dry to extremely dry water balance during the spring and summer months (ca., 407 mm of rain inputs in 2020 versus 115 mm in 2021), as shown in Table 2 and Figure 2. The first quarter of the year 2021 was characterized by low-intensity precipitation events, clear sky, advection of cold air, and thermal inversion conditions at dawn, with frequent and intense frost in the late period (April). In addition, in late March and early April, a few rainfall events were recorded, coinciding with the beginning of fruit growth (Figure 2). However, rainfall from May to September did not exceed 50% of the climatological average. Thus, there was a marked water deficit, with a sharp reduction in the available soil water content, from April to September, with an average PD of 144 mm in 2021 versus 238 mm in 2020 (Table 2 and Figure 2). Nevertheless, compared to 2020, slightly lower temperatures and smaller heat units were recorded during winter 2021, and this resulted in a slight delay in phenological succession from bud burst until the end of flowering for ‘Tuono’ and a longer bloom for ‘Arrubia’ and ‘Texas’ (Figure 1 and Table 2). Conversely, from April to September, the heat units were high, although it did not significantly affect the duration of phenological stages. 

**Figure 3:**
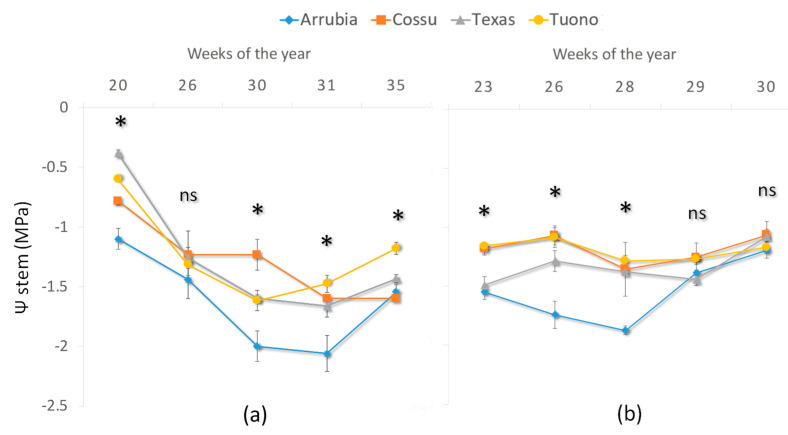
Patterns of midday stem water potential in each cultivar during the growing seasons 2020 (**a**) and 2021 (**b**). Values are mean ± standard error. Significant differences for *p*-value of 0.05 are represented by * and ns indicates not significant differences among cultivar.

The irrigation scheduled during the period from May to September provided 372 mm of water in the season of 2020 and 495 mm in 2021 (Table 2). The irrigation events were divided into 2 weekly irrigations, with a duration of 6 and 9 h, for a total amount of about 218 m^3^ ha^−1^ each week. In 2020, the weekly intervals were kept constant throughout the irrigation season. In 2021, due to the early and prolonged deficit of the water balance, a third irrigation event per week was added in July and August, for a total number of 20 h of irrigation per week to restore approximately 29.1 mm of water per week. From pit-hardening to the beginning of the nut growth stage, about 150% and 170% ETc were returned in 2020 and 2021, respectively (Table 3). A gradual increase in deficit occurred in the following stages and only 70% ETc and 80% ETc were returned, respectively, in 2020 and in 2021, by irrigation and precipitation events (Table 3). In 2020, at the beginning of kernel filling, the irrigation supply below the actual water plant consumption led to an increase in the deficit (55% and 65% ETc during kernel filling and until hull split, respectively) and to a moderate to severe water stress status of the plants (Table 3 and Figure 3). In 2021, the early establishment of soil water deficit required an increase of 25% in the weekly re-watering volume (about +35 mm), so that a mild to moderate water stress condition could be maintained from the beginning of the kernel filling stage until harvest (to, ca., 80% ETc). Thereafter, irrigation stopped as the water balance became positive, ensuring good soil water availability during bud differentiation.

The patterns of Ψ_stem_ during the irrigation season shown in Figure 3 reflect the differences observed between years with regard to soil water reservoir, being initially high in 2020 and already reduced at the same period of the following year, and differences among Sardinian and non-Sardinian varieties, with typical values of a mild stress condition in ‘Cossu’ and ‘Arrubia’ (ranging from −0.8 and −1.1 MPa, respectively) and mild to absent water stress in ‘Tuono’ and ‘Texas’ (with Ψ_stem_ ranging from −0.3 and −0.6 MPa, respectively). The Ψ_stem_ decreasing trend continued in 2020 until the first autumn rainfall, with significantly lower values in ‘Arrubia’ than in the other varieties (with minimum values of about −2.0 MPa vs. −1.5 in ‘Tuono’ and ‘Texas’ and −1.2 to −1.6 MPa in ‘Cossu,’ respectively). The decreasing trend in Ψ_stem_ and the differences between ‘Arrubia’ and the other varieties were also observed during the irrigation season of 2021 (the more negative values varying among −1.9 MPa in ‘Arrubia’ and −1.3 MPa to −1.4 MPa in the other varieties). However, the Ψ_stem_ decreasing trend was inverted from week 28 onward when, as the irrigation supply increased from 21.8 mm to 29.1 mm, distributed in 3 watering supplies per week, plant water status was progressively restored to reach a moderate and then mild stress condition in all varieties.

### 3.2. Cultivar Physiological Performance and Yield Responses

#### 3.2.1. Water Relations and Photosynthetic Activity

**Figure 4:**
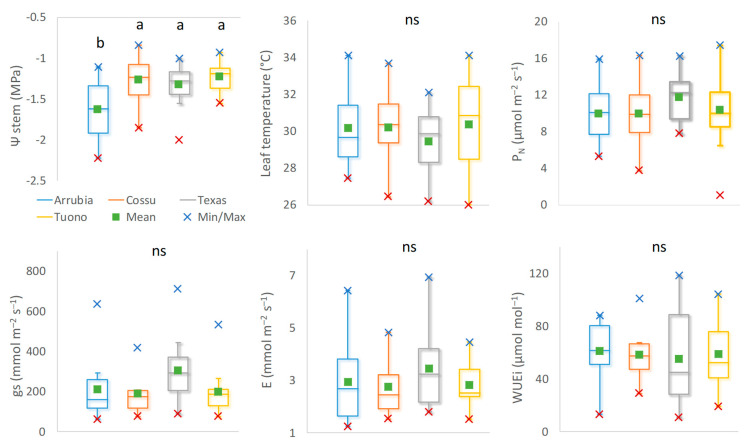
Boxplot and one-way ANOVA significance of differences among varieties of observed midday stem water potential, leaf temperature, net photosynthesis, stomatal conductance, transpiration, and intrinsic water use efficiency rates during nut growing stage. Data are the mean of tree replicates from the 11 dates of measurements (n = 55). Box limits are the first and third quartile. Different lowercase letters indicate significant differences in mean values among cultivar and ns indicates not significant differences among cultivar.

**Figure 5:**
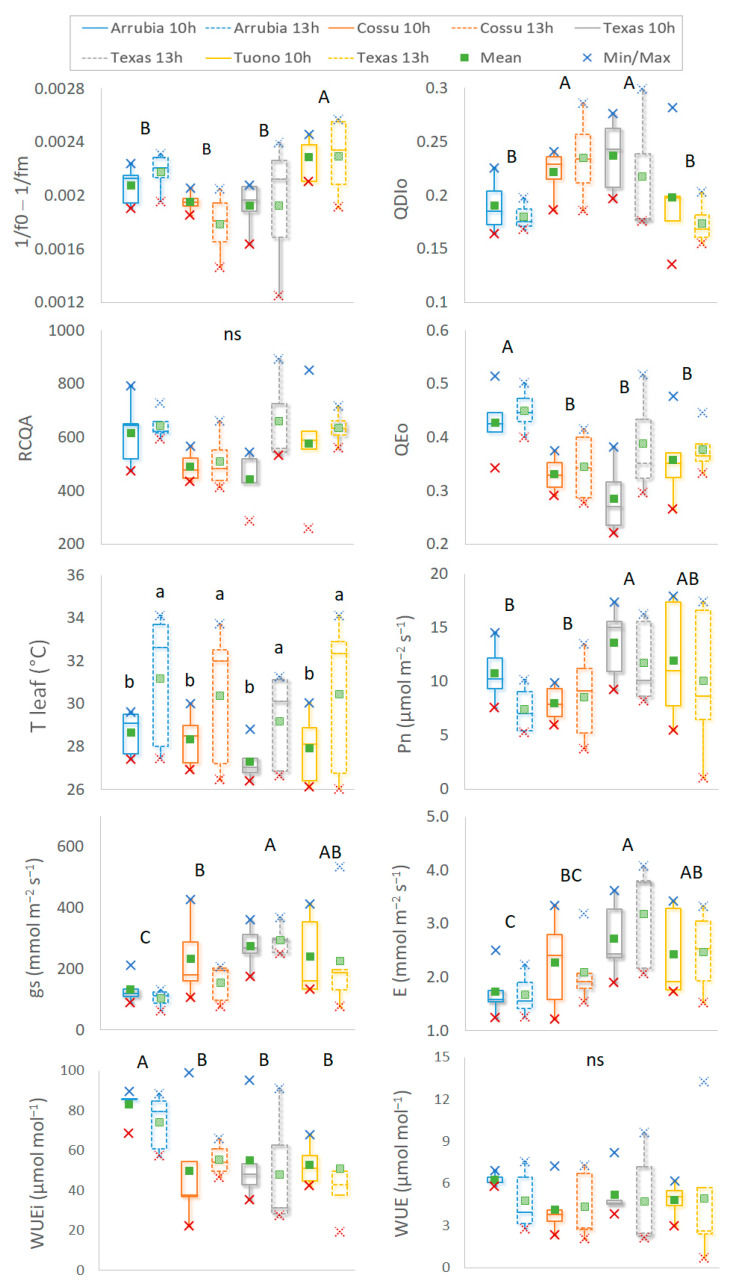
Boxplot and two-way ANOVA significance of the effects of cultivar and daytime measurement for midmorning and midday leaf photochemical and photosynthetic performance during nut growing and ripening stages in the two-year study period. Data are the mean of tree replicates, from 4 dates of measurements (n = 20). Box limits are first and third quartile. Full-line and dotted-line boxplots represent, respectively, data collected during mid-morning and midday. Different capital letters indicate significant differences among cultivar, different lowercase letters indicate significant differences between years and ns indicates not significant differences among cultivar.

The whole midday plant water status and leaf gas exchange data distribution and skewness showed important differences among cultivar that help characterize cultivar physiological responses under water stress conditions (Figure 4). ‘Arrubia’ showed a significantly lower Ψ_stem_ throughout the season but also a larger interquartile range as compared to the other cultivar. Though the differences in Ψ_stem_ among the other three cultivar were not significant, ‘Tuono’ and ‘Texas’ displayed high values and a lower dispersion with respect to both the two Sardinian cultivar. Despite the differences in plant water status, leaf gas exchange performance were similar, both for the average values and interquartile data distribution, especially for net photosynthetic assimilation. The stomatal conductance (gs) ranged within similar values in the four varieties during the seasons and only a slightly larger variation and higher values were observed in ‘Texas’, as compared to ‘Cossu’ and ‘Tuono’, for which the stomatal resistance to water transport reached, on average, lower values and higher stability across the seasons. Consequently, higher E rates were also reached in ‘Texas’ leaves as compared with ‘Tuono’ and ‘Cossu’. However, ‘Arrubia’ leaves were also able to transpire at higher rates and the variation in the measured values was much higher than those of ‘Tuono’ and ‘Cossu’, despite the significantly lower water potential in the plants. On the whole, ‘Arrubia’ plants were able to retain higher mean and interquartile values of WUEi, and the highest and the lowest absolute values were measured in ‘Texas’. Though the differences among means were not statistically significant, a higher stability of WUEi in response to water status and evaporative demand along the seasons was observed in ‘Cossu’. Both the two-way ANOVA and whisker plots of leaf photosynthetic activity and primary photochemistry showed a main effect of cultivar on leaf physiological performance, while the effect of the time of the day during which the measurements were taken, at midmorning or midday, was not statistically significant when the whole data collected during fruit growth and ripening were considered (Figure 5). As the quantum yield can be well addressed by the indicator of functional PSII [51] units and as φ_DIo_ is mathematically the inverse of the φPo, this variable is not included in Figure 5. Higher interquartile variability was found at midday in what concerns maximum quantum yield (either represented by 1/Fo − 1/Fm or by φPo), energy dissipation, and functional PSII units along the season, particularly in ‘Cossu’, ‘Texas,’ and ‘Tuono’.

This can be explained by the higher ranges within which leaf temperature varied at midday hours across the seasons, when the evaporative demand, and especially heat and drought stresses, can reach higher maximum values on clear sky summer days or remain low and quite similar to midmorning values during cloudy days. In fact, water stress and high temperatures were the limiting factors affecting photosynthesis performance in this study, and this also evidenced the lower interquartile variation from midmorning to midday measurements in the ‘Arrubia’ plants that presented a lower water potential over the season. Though average leaf temperatures were not significantly different among varieties, ‘Tuono’ leaves displayed slightly higher values. The main cultivar effect regarded 1/F_o_ − 1/F_m_, φ_Po_, φ_DIo_, and φ_Eo_. Under the environmental conditions of the study, ‘Tuono’ leaves were able to yield significantly greater photochemical activity and lower energy dissipation, while the RCDA and φ_Eo_ ranged between tighter and high limits, probably due to the lower energy dissipation and, hence, higher thermal stress [51,52,54]. ‘Arrubia’ plants showed the highest φ_Eo_ during the primary steps of photochemical reactions and, though φ_Po_ and φ_DIo_ mean values were not statistically different from those of ‘Cossu’ and ‘Texas’, ‘Arrubia’ showed high interquartile data of maximum quantum yield for photochemistry and functional PSII units, closer to those of ‘Tuono’. The higher photochemical performance of ‘Tuono’ was followed by a high net photosynthetic assimilation, though the interquartile data fluctuated among a wider range during the season. A higher Pn was observed also in ‘Texas’ leaves and, in this case, the values were significantly higher than those of ‘Arrubia’ and ‘Cossu’. Similarly, gs and E were higher in ‘Tuono’ and ‘Texas’ but only significantly higher from ‘Cossu’ and ‘Arrubia’ in ‘Tuono’ leaves (Figure 5). 

**Figure 6:**
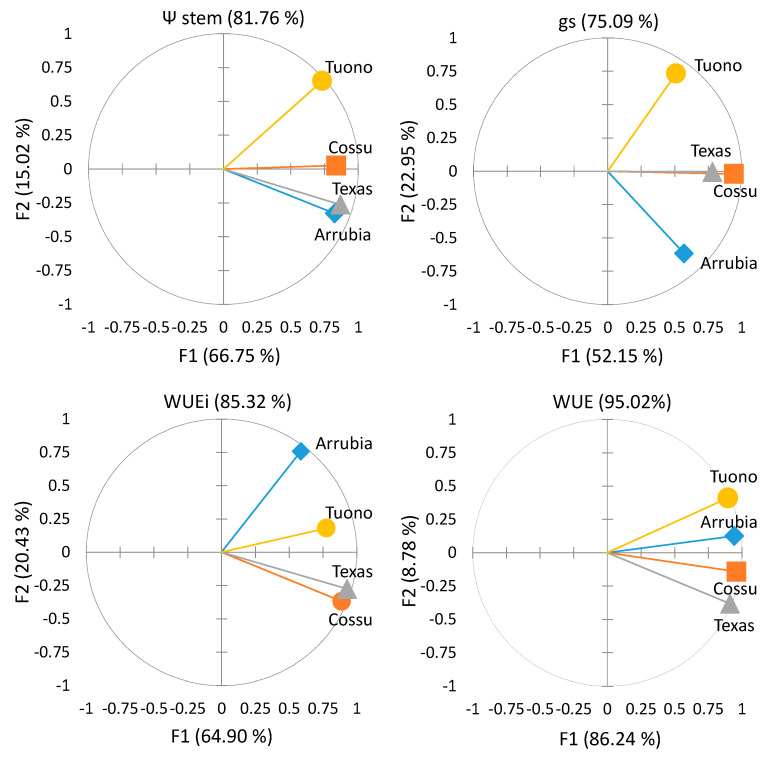
Principal Component Analysis (PCA) plots showing the variation among the four cultivar in terms of midday stem water potential (Ψ_stem_), stomatal conductance (gs), intrinsic water use efficiency (WUEi), and transpiration efficiency (WUE) during nut growth stage of the two-year study period. Data are the mean of each tree replicate from 11 dates of measurements (n = 55).

The lower gs in ‘Arrubia’ allowed for a significantly higher intrinsic water use efficiency across the season and the transpiration losses were also significantly lower, yet the efficiency for transpired water during photosynthesis was not significant different among varieties, because WUE varied within lower minimum values but similar maximum limits in the other three varieties across the season. The differences among cultivar concerning these physiological responses were further evaluated using principal component analysis (PCA), in order to summarize and compare the variation and the strength of midday performance variation in the four cultivar (Figure 6). The PCA plot of midday Ψ_stem_ and leaf gas exchange performance showed a different variation in water potential and gs on ‘Tuono’ compared to the other cultivar. ‘Arrubia’ and ‘Texas’ Ψ_stem_ varied similarly_,_ but the stomatal behavior and WUEi of ‘Arrubia’ were much different, also from those of ‘Cossu’ and ‘Texas’, which showed similarity in WUEi and WUE. 

The variations in photosynthetic performance and plant water status were explained by two main factors (Figure 6). The first factor explained about 67%, 52%, 65%, and 86% of the variation in Ψ_stem_, gs, WUEi, and WUE, respectively, and the second factor explained only 15%, 23%, 20%, and 9% of these variations in all cultivar. The gas exchange performance and water potential of these plants were highly correlated with factor 1, which can be associated with the water deficit and evaporative demand. Factor 2 could be described as a varietal response strength as it was only highly correlated with ‘Arrubia’ Ψ_stem_ and with ‘Arrubia’ and ‘Tuono’ gs, while the explanatory strength of the second factor was negligible for ‘Cossu’ and ‘Texas’. These results confirm that soil water deficit and atmosphere evaporative demand are the most important factors driving gas exchange performance of almond trees in Mediterranean climates, but the response of each variety to the fluctuations in heat and water stresses are also affected by adaptive mechanisms that determine an important and different resilience, in terms of photosynthetic activity and resistance to water loss along the plant, among varieties. The analysis of plant water status and photosynthetic and photochemical performance evidenced a great plasticity of almond trees in adapting leaf physiological performance to drought and hot conditions during fruit development and ripening stages under Mediterranean environments, where prolonged water and heat stresses are repeatedly experienced during the summer. This capacity was particularly evident in ‘Arrubia’ and ‘Tuono’. Cultivar Tuono showed a constantly high photochemical performance and, on average, leaf photosynthetic activity reached the highest values and remained quite high under restricted stomatal aperture. In addition, ‘Texas’ presented an elevated net photosynthesis across the season, yet the photosynthetic apparatus evidenced lower functional performance and less constancy of the variables that represent photochemical yield, heat dissipation, and the integrity and efficiency of the primary photosynthetic system. Among all cultivar, ‘Arrubia’ exhibited a higher leaf photosynthetic and photochemical efficiency under moderate water stress. In this variety, the stomatal control over transpiration was significantly higher and, even at lower water potential, leaves were able to perform at elevated rates, always maintaining high efficiency in what concerns heat dissipation and photochemical and photosynthetic activity, particularly when compared to ‘Cossu’ and ‘Texas’. Considering all of the leaf physiological variables, in the conditions of this study, cultivar Cossu revealed a lesser capability to adapt to water and heat stresses, though the average values were within the 50% interval of the other varieties for most of these variables.

#### 3.2.2. Yield and Water Use Efficiency

Yield alternation was marked in 2021 as compared to 2020 and, besides the significant effect on yield per plant in all four varieties, season also influenced kernel weight, which was higher in the second season, due to a very low fruit load, hence the reduced competition within the canopy. However, the differences in fruit yield and yield components were also highly significant among varieties across the two seasons (Table 4). 

‘Cossu’ plants yield low fruit number per plant in both seasons, but especially in 2021. Such reduced crop load leads to a feeble source–sink balance and this has most likely contributed to the low photosynthetic performance of this variety during fruit growth and ripening stages. In effect, though the differences among the other three cultivar were not significant in terms of yield per plant, ‘Texas’, ‘Arrubia,’ and ‘Tuono’ yielded higher nut weight, also considering that in ‘Texas,’ a significantly higher percentage of double kernels are produced, regardless of the season alternation effect. Interestingly, the two Sardinian cultivar present significantly higher fruit weight and also higher nut weight, especially ‘Arrubia’. Again, the higher fruit load, nut weight, kernel length, and width in the latter cultivar were able to determine an intense sink effect of fruits on net assimilation, water potential, and photo-assimilation translocation rates through the plant, thus explaining the higher performance and leaf gas exchange efficiency observed in this study. The effect of season was varietal-dependent in what concerns nut weight and kernel width: in the two local cultivar, the first parameter was higher in 2020 compared to 2021, while the second parameter was higher in 2020 only in ‘Arrubia’. No cultivar—season interaction effect was observed in fruit weight and percentage of double kernels, and for the other parameters, yield per plant and kernel length, the interaction effect was proportional to that of varietal differences.

**Figure 7:**
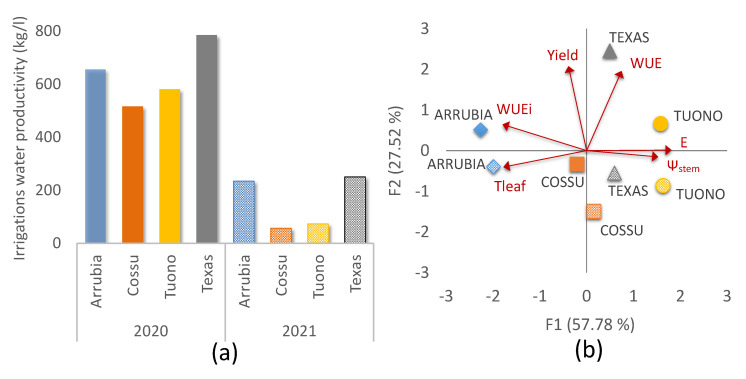
Irrigation water productivity in the four almond cultivar during the two years of study (**a**); PCA biplot showing the distribution, strength, and relationship among cultivar and physiological variables characterizing plant and leaf water status, temperature, and water use efficiency during the study period (**b**). Full and dotted symbols represent, respectively, 2020 and 2021 data.

In order to better understand the implications of the differences among cultivar in terms of leaf physiological activity, water stress adaptive capacity, and productivity in the environmental context of this study, irrigation water productivity (IWP) was calculated and the variables that better describe the efficiency of a plant in the use of water during photosynthesis under water stress were represented in a bi-dimensional space (Figure 7).

As expected, the IWP in season 2020 was significantly higher than in 2021. This was due to the strong reduction in yield per plant that occurred in 2021. The increase in watering supplies during ripening in 2021 to face heat and drought stress at these stages induced a better plant water status, and a positive effect of this increased irrigation volume may effectively improve bud differentiation and, hypothetically, could result in a higher IWP in the next season yield. The biplot in Figure 7b shows that, with a moderate water deficit, Ψ_stem_, E, Tleaf, and WUEi vary strongly within factor 1 and, for increases in leaf temperature within the conditions of our study, plant water potential and transpiration rates decrease, yet WUEi is still able to increase due to a high photosynthetic activity, as described previously, and a strong stomatal control. Two of the four cultivar were strongly correlated with these variables during the two-season trial, ‘Arrubia’ and ‘Tuono’. Conversely, ‘Texas’ and ‘Cossu’ were weakly correlated with factor 1 and mostly reflected the effect of yield and WUE in 2020, for ‘Texas,’ and low yield in 2021 for ‘Cossu’.

## 4. Discussion

The winter chilling during 2020 resulted in an overall advance in the phenological succession compared to 2021. For many almond varieties, including those studied here, quite few chilling units are required for breaking dormancy and, thus, heat requirements for flowering and successive growth are reduced [23]. This fact explains the shorter duration of the phenological stages from bud burst to the end of flowering observed in 2020 on three of the four varieties, with the exception of ‘Cossu’. In fact, the substantial differences in phenological courses among cultivar and seasons concern almost exclusively these early stages, as the thermal regime in the successive months is frequently optimal and rarely represents a limiting factor for the phenological rhythms of almond orchards in Mediterranean environments. When comparing the phenology of the four varieties during the two years, less variability in bud burst timing and in the duration of flower development and bloom were denoted in the Sardinian varieties. These two varieties are characterized by an early bloom, while ‘Texas’ and ‘Tuono’ present, respectively, late and medium-late bloom [3,12,55]. However, the higher thermal summation during the first quarter of the year 2020 induced an anticipation of budburst and shortened the duration of bloom to fruit set stages in ‘Tuono’ and ‘Texas’, respectively. A slight anticipation of flowering in ‘Arrubia’ and ‘Cossu’ compared to ‘Tuono’ has been reported previously [12] and a longer duration of this stage in ‘Texas’ compared to other varieties was previously documented [55]. Bloom also took place slightly faster in ‘Arrubia’ in 2020 but, overall, the duration of the vegetative cycle remained unchanged in both ‘Arrubia’ and ‘Cossu’, which showed a lower sensitivity to the inter-annual variation in temperature during the first phenological stages, especially when compared to ‘Tuono’. In addition, it is important to underline that the frost events that occurred in January and February 2021 severely impaired fruit set, resulting in a sharp decrease in whole orchard yields. The effect of seasonal weather, namely of extreme cold or heat waves, remains crucial for flowering duration and pollination effectiveness [56], but besides the great influence of the meteorological conditions, the self-fertility of cultivar, such as ‘Tuono,’ and a high number of flowers, as in ‘Texas’, may foster the success of pollination and fruitfulness [57,58,59]. 

**Figure 8:**
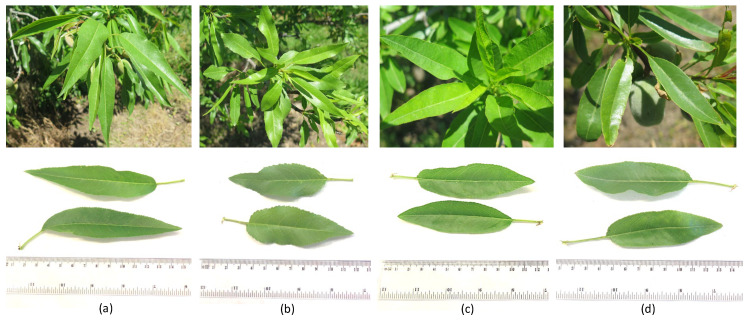
Example of adult leaves in spurs of cultivar Arrubia (**a**), Cossu (**b**), Tuono (**c**), and Texas (**d**), showing different blade shape and size.

During both years, the orchard was exposed to mild to moderate water deficit conditions during fruit development and ripening stages, but in 2021, the water deficit was established earlier, after fruit set, due to the lower rainfall as compared to 2020, determining a lower Ψ_stem_ at the pit-hardening stage (about—1.3 MPa in 2021 against −0.7 MPa in 2020). Differences in Ψ_stem_ declining rate among varieties were observed and a gradual development of water stress may allow for progressive acclimation, preventing excessive defoliation and plant mortality under severe drought [17,21]. However, the most efficient drought-adaptive response observed in our study was the strict stomatal control over transpiration under lower plant water content shown by ‘Arrubia’ while maintaining photosynthetic levels. In effect, varietal responses to water stress during fruit development and ripening stages were different, both in the regulation of water status, leaf gas exchange, and leaf primary photochemical efficiency. ‘Arrubia’ maintained a lower Ψ_stem_ compared to the other varieties, while sustaining high photosynthetic activity through strict stomatal control and reduced transpiration losses. Besides the greater water use efficiency, this variety displayed an efficient use of the photonic energy during photosynthesis and was able to maintain leaf temperature and efficiently dissipate excessive energy from leaf photosynthetic apparatus. ‘Texas’ also showed a similarly high primary photochemical efficiency but under a milder water status. These results indicate a great variability among varieties in terms of the plasticity to cope with soil water scarcity and demonstrate a diverse capacity to adapt to prolonged drought and heat stresses during fruit development. Previous studies [28] reported differences among almond varieties concerning leaf water and osmotic potentials and related these differences with leaf relative water content, suggesting that leaf anatomical differences, namely a higher cell wall rigidity, allowed for keeping a higher apoplastic water content and to prevent turgor-excessive dehydration and metabolism alteration. In addition to the higher leaf gas exchange efficiency observed in ‘Arrubia’ and ‘Texas’ plants under moderate to mild water stress conditions, higher yields were also reached in these varieties compared to both ‘Cossu’ and ‘Tuono’. It is also important to underline that, despite being a self-incompatible variety [60], ‘Texas’ was able to present higher yield levels in 2020, regardless of a rapid occurrence of bloom and fruit set, which indicated a high fruit set rate and confirms the closer relationship of yield to the abundance of flowers rather than to the ratio of flowers that fruit set to total number of flowers, as previously demonstrated [59]. In fact, fruit set and bud density are the main factors influencing yield [60,61] and, in our trial, this higher yield depended upon an elevated fruit number as the fruit weight of ‘Texas’ plants was lower than those of ‘Cossu’ and ‘Tuono’, which showed a much lower yield. The alternation of yield levels between the two seasons was marked, showing that sufficient soil water during fruit set and pit hardening is the most important factor affecting crop production at this stage, namely kernel formation and development. Nevertheless, as compared to ‘Arrubia’ and ‘Texas’, ‘Cossu’ and ‘Tuono’ kept the same lower yield proportion that was observed in 2020, therefore ascribable to a more limited acclimation response to water stress rather than to the interannual differences in the evaporative demand and soil water availability. Besides ‘Arrubia’, which showed both a high productivity and an ability to cope with water scarcity, ‘Texas’ also physiologically well acclimated to the site environmental conditions, displaying both high flower induction and high fruit set capacity. Moreover, the high fruit set rate in ‘Texas’ played an indirect important role on net photosynthetic performance and efficiency during fruit growth and ripening stages, as a higher number of growing fruits exerted an effective sink strength for photosynthates translocation and leaf metabolism. On the other hand, the self-fertility of ‘Tuono’ did not promote a higher yield. Previous studies concerning almond cultivar yield performance in Sardinia reported high yield levels of ‘Tuono’ compared to other highly performing varieties cultivated in most Mediterranean environments [34]. However, in our study, this cultivar showed high stomatal conductance and transpiration rates, as compared to both ‘Arrubia’ and ‘Texas’. Therefore, when the water deficit conditions are protracted, a higher variety sensibility determines a lower net productivity across the season. Despite being considered mainly auto-incompatible, ‘Arrubia’ maintained a higher yield over the season [62]. The greater performance of both ‘Arrubia’ and ‘Texas’ compared to other varieties have also been reported by other authors [15]. However, the results of our study suggest that different factors were responsible for the differences in crop yield and leaf performance in these two varieties. A higher water use efficiency coupled with highly efficient control over transpiration in ‘Arrubia’ allowed for maintaining a high metabolic performance and fruit nurturing, while fruit sink strength promoted a higher leaf metabolism in ‘Texas’. These significant differences in plant physiological performance and acclimation capacity were also highlighted by a low defoliation rate across the season in those two varieties compared to ‘Tuono’ and especially to ‘Cossu’. Other than the feeble fruit sink strength of ‘Cossu’ plants, the high leaf abscission rate of this cultivar is influenced by botanical and anatomic traits such as dominant shoot type (vegetative shoots and spurs) and leaf characteristics (Figure 8, Table A2), namely size and roughness (higher in ‘Arrubia’ and ‘Texas’), that affect leaf hydraulic conductance, a major determinant on stomata regulation [28,59,63]. Moreover, previous work has demonstrated that a greater leaf area in the previous year induces spur survival into the next year, with a higher probability for the spur to bear flowers [59]. In addition, [64] suggested that the source–sink balance in the spur itself is a determinant for a regular yield, as the spur seems to present a semi-autonomous behavior for carbohydrates use during fruit growth and development. 

## 5. Conclusions

In this study, the physiological and yield performance of four field-grown almond cultivar from different Mediterranean climate regions, progressively subjected to deficit irrigation during fruit development and ripening, were studied in order to evaluate the cultivar acclimation response to water stress. The two Sardinian varieties, Arrubia and Cossu, showed marked differences in water stress tolerance, photosynthetic and photochemical activity, and crop yield. ‘Arrubia’ showed an intrinsically greater physiological acclimation to water stress and ability to reach higher yields, while in ‘Cossu,’ plant performance were marginal and intense defoliation was observed during the season. The defoliation allowed for maintaining higher plant water content, which helps prevent plant mortality under prolonged drought under a non-irrigated and/or low-soil-fertility context. However, a pronounced discontinuity between leaves and photosynthate translocation deeply compromises orchard productivity and, hence, crop income. Under the environmental conditions of the study, the self-fertile cultivar Tuono also showed low acclimation and adaptation capacity to progressive water stress, with a rapid increase in stomatal conductance, low yield, and low water use efficiency. The Californian cultivar Texas has acclimatized well to the site conditions and to water stress but, in this case, the main factor responsible for the physiological acclimation was the crop load, which promoted an effective sink strength for an intense and continuous translocation from the leaves through the plant. The important role played by specific variety anatomical traits affecting plant physiological performance was also evident in our study. In fact, leaf ultra-structure, dimensions, and roughness may be key traits of almond plant water stress responses and for the efficient endurance of the photosynthetic apparatus, influencing leaf stomatal conductance, transpiration, and cell turgor. Overall, the distinct varietal acclimation to environmental stresses much depended upon these traits, regardless of self-fertility vs. semi-sterility characteristics of the studied varieties. The results of our study highlight the importance of recognizing and characterizing the relationships among the traits of almond varieties that affect plant performance under drought, in order to better assist planting choices and orchard irrigation management for a given environmental context to reach a specific crop production target.

## Figures and Tables

**Table 1:** Direct chlorophyll fluorescence of PSII main and derived variables.

Direct Fluorescence Variables	Equation	Reference
Maximum yield of primary photochemistry of PSII	φPo = Fv/Fm	[52]
Variable fluorescence	Fv = Fm − Fo	
Linear indicator of functional PSII units	1/Fo − 1/Fm	[51]
Maximum water-splitting efficiency	Fv/Fo	[52]
Quantum yield for electron transport	φEo = (Fv/Fm) × (1 − Vj)	
where Vj is the relative variable fluorescence at J-step (2 ms)	Vj = (F2ms − Fo)/(Fm − Fo)	
Quantum yield for energy dissipation	φDIo = 1 − φPo	
Density of the reaction centers	RCQA = φPo × (ABS/CSm) × (Vj/Mo)	
where Mo is the initial slope (in ms^−1^) of the fluorescence curve with respect to F300	Mo = 4 × (F300 μs − Fo)/(Fm − Fo)	
and ABS/CSm is the absorption of photon flux by antenna chlorophyll molecules of active and inactive reaction center of PSII per excited cross-section of leaf area	ABS/CSm	

**Table 2:** Average meteorological conditions and irrigation supply during the study seasons.

Quarter	T Maximum (°C)		T Minimum (°C)		PP (mm)		ET0 (mm)		PD (mm)		Irrigation (mm)	
	2020	2021	2020	2021	2020	2021	2020	2021	2020	2021	2020	2021
January–March	16.2	15.2	7.7	6.9	83	182	110	118	283	323	-	-
April–June	23.7	22.9	13.3	12.7	159	77	360	355	212	144	175	175
July–September	29.7	30.3	18.9	19.4	248	39	405	375	26	0	197	320
October–December	18.2	18.8	10.3	10.5	310	185	100	94	276	42	-	-
Annual	22.0	21.8	12.6	12.5	800	482	975	942	199	126	372	495

**Table 3:** Orchard water balance, deficit irrigation strategy, and midday plant water status comparison among four cultivar during the growing seasons. The gradients of increasing plant water stress in the four cultivar are represented by the gradients of color intensity, respectively, from dark blue to light blue, and to light and dark red.

Season	Phenological Stage	Weeks of the Year	Kc	ETc	PP-ETc	Returned ETc (%)	Plant Water Status Gradients			
							Arrubia	Cossu	Texas	Tuono
2020	Fruit set—Pit hardening	14–18	0.75	59.2	39.4	-				
	Pit hardening—Nut growth	18–22	0.75	98.9	−57.9	150				
		22–27	0.95	141.8	−123.0	70				
	Kernel filling—Hull split	27–31	0.95	164	−163.6	55				
		31–36	0.95	136.6	−135.2	65				
	Harvest—Bud differentiation	36–40	0.85	75.1	171.5	-				
2021	Fruit set—Pit hardening	13–17	0.75	61.7	−59.7	-				
	Pit hardening—Nut growth	18–22	0.75	91.4	−38.2	170				
		23–26	0.95	143.3	−142.7	80				
	Kernel filling—Hull split	26–31	0.95	148.6	−146.2	80				
		31–35	0.95	127.2	−127.0	70				
	Harvest—Bud differentiation	35–39	0.85	72.3	−72.3	-				

**Table 4:** Fruit yield and yield compounds during the study seasons. Values are the mean ± standard error and two-way ANOVA of cultivar, season, and interaction effects. Different capital letters indicate significant differences among cultivar and different lowercase letters indicate significant differences among seasons.

CV	Season	Yield (Kg/plant)	Fruit Weight (g)	Nut Weight (g)	Kernel Weight (g)	Kernel Length (cm)	Kernel Width (cm)	Double Kernel (%)
Arrubia	2020	6.1 Aa	12.89 A	7.47 Aa	1.42 b	2.66 Ab	1.73 Aa	4.4 B
	2021	2.9 Ab	10.91 A	7.36 Ab	1.58 a	2.76 Aa	1.66 Ab	3.3 B
Cossu	2020	4.8 Ba	10.28 A	6.00 Bb	1.41 b	2.24 Bb	1.60 Bb	2.2 AB
	2021	0.7 Bb	11.83 A	6.80 Ba	1.59 a	2.46 Ba	1.70 Ba	11.1 AB
Tuono	2020	5.4 Aa	8.04 B	4.87 Cb	1.48 b	1.84 Bb	1.35 Db	0 Cc
	2021	1.9 Ab	9.16 B	5.46 Ca	1.71 a	2.71 Ba	1.52 Da	0 Cc
Texas	2020	7.3 Aa	7.54 B	4.75 Cb	1.26 b	2.43 B	1.46 Cb	12.2 A
	2021	3.1 Ab	8.64 B	5.34 Ca	1.62 a	2.44 B	1.58 Ca	11.1 A
Sig.	Cultivar	0.001	0.001	0.001	0.127	0.001	0.001	0.001
	Season	0.005	0.712	0.003	0.001	0.001	0.005	0.1
	Interaction	0.016	0.67	0.019	0.011	0.001	0.001	0.74

## Data Availability

The data presented in this study are available from the corresponding author on reasonable request.

## Supplementary Materials

The following supporting information can be downloaded at: https://www.mdpi.com/article/10.3390/plants12051131/s1, Figure S1: Experimental design, identifying blocks, and variety replicates distribution along the rows of the almond collection orchard (a); aerial image of the orchard (b).

## Appendix A

**Table A1:** Selected soil textural and hydraulic properties.

Texture Class	Depth (cm)	Particle Size (% wt.)			Organic Matter	pH	Saturation	Field Capacity	Wilting Point
		Sand	Loam	Clay	(%)		(% vol)		
Clay-loam	0–20	42.7	27.1	30.2	2.33	7.35	46.6	32.3	12.8
Sandy-clay-loam	20–50	56.2	18.0	25.8	1.78	8.58	40.8	26.1	10.1
Average values	0–50	49.5	22.6	28.0	2.1	8.0	43.7	29.2	11.5

**Table A2:** Average dimensions and leaf area of fully expanded leaves of the four varieties. Values are mean (n = 15) ± standard error. Different lowercase letters indicate significant differences among cultivar.

	‘Arrubia’	‘Cossu’	‘Tuono’	‘Texas’	Significance
Leaf length (cm)	11.1 ± 0.34 a	7.9 ± 0.28 c	9.06 ± 0.32 b	8.8 ± 0.26 b	0.001
Leaf width (cm)	2.7 ± 0.07	2.7 ± 0.10	2.7 ± 0.10	2.8 ± 0.08	0.420
Area (cm^2^)	93.4 ± 4.48 a	66.8 ± 3.82 c	77.4 ± 2.94 bc	79.1 ± 3.4 b	0.001
