# Supplementary material for: Almond Tree Adaptation to Water Stress: Differences in Physiological Performance and Yield Responses among Four Cultivar Grown in Mediterranean Environment
Source: Plants (Basel). 2023 Mar 2;12(5):1131. doi: 10.3390/plants12051131 (PMC10004802; doi:10.3390/plants12051131)
Supplement: Supplementary file 1 [file plants-12-01131-s001.zip › plants-2199193-supplementary.pdf]

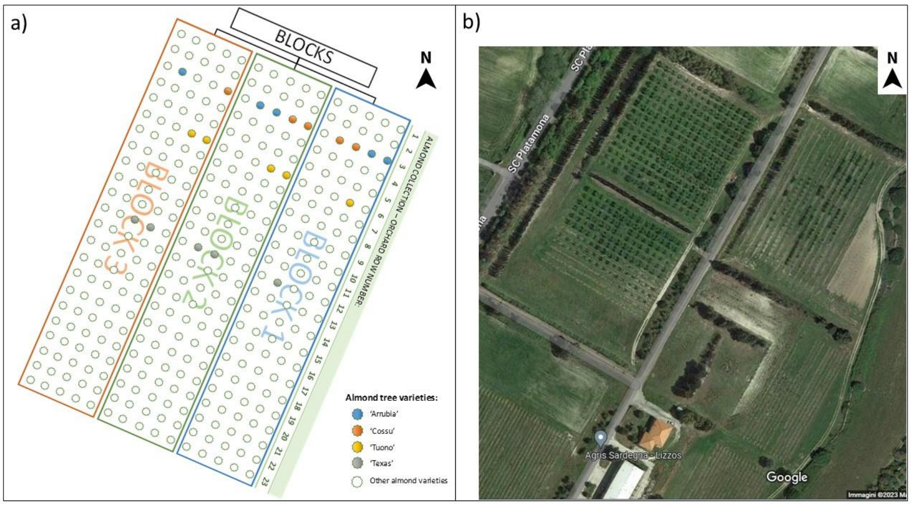

Figure S1: Experimental design, identifying blocks, and variety replicates distribution along the rows of the almond collection orchard (a); aerial image of the orchard (b).
